# [Corrigendum] Chloroquine potentiates the anticancer effect of sunitinib on renal cell carcinoma by inhibiting autophagy and inducing apoptosis

**DOI:** 10.3892/ol.2024.14403

**Published:** 2024-04-17

**Authors:** Man-Li Li, You-Zhi Xu, Wen-Jie Lu, Yong-Huai Li, Shi-Sheng Tan, Hong-Jun Lin, Tian-Ming Wu, Yan Li, Si-Ying Wang, Ying-Lan Zhao

Oncol Lett 15: 2839–2846, 2018; DOI: 10.3892/ol.2017.7635

Following the publication of the above article, an interested reader contacted the Editorial Office to inform us that, in Fig. 4D on p. 2845 showing the results of TUNEL assay experiments, the ‘Control’ and ‘CQ’ data panels appeared to be strikingly similar, such that these data may have been derived from the same original source, where the data were intended to show the results from differently performed experiments.

The authors have consulted their original data, and were able to identify where the error occurred in terms of the data selection for Fig. 4B. The corrected version of Fig. 4, showing the correct data for the ‘Control’ panel in Fig. 4D, is shown on the next page. All the authors approve of the publication of this corrigendum, and the authors are grateful to the Editor of *Oncology Letters* for granting them the opportunity to publish this. The authors also apologize to the readership for any inconvenience caused.

## Figures and Tables

**Figure 4.**
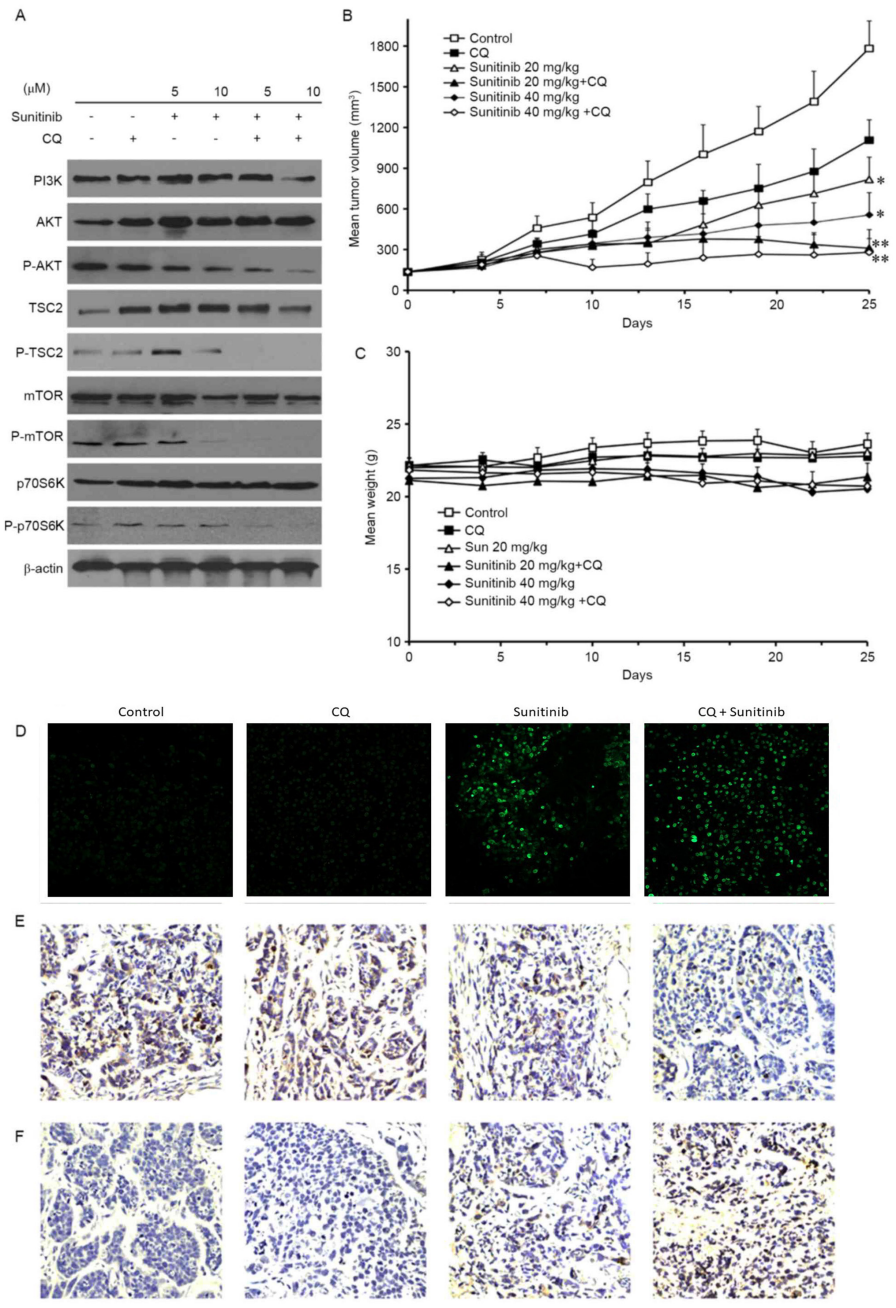
Sunitinib induces autophagy by inhibiting the Akt/mTOR/p70S6K signaling pathway, and may inhibit the growth of OS-RC-2 tumors *in vivo*. (A) Protein expression of PI3K, AKT, p-AKT, TSC2, p-TSC2, mTOR, p-mTOR, p70S6K and p-p70S6K in OS-RC-2 cells. (B) The inhibitory effect on the proliferation of OS-RC-2 cells established in nude BALB/c mice following CQ (20 mg/kg/day intravenously), sunitinib (40 mg/kg/day orally) or both. The data were expressed as the mean ± SD. (C) Mouse body weight was not significantly altered following sunitinib and CQ co-treatment. (D) A terminal dexynucleotidyl transferase-mediated dUTP nick end labeling assay was performed to measure the induction of apoptosis. (E) Tumor cell proliferation in different groups was analyzed by the staining of phosphorylated histone H3. (F) Tumor cell autophagy in different groups was analyzed by LC3-II staining. Data represent the means ± SD or are representative of ≥3 independent experiments (magnification, ×200). *P<0.05 and **P<0.01 compared with control. PI3K, phosphoinositide 3-kinase; p-, phosphorylated; TSC2, tuberous sclerosis complex 2; mTOR, mechanistic target of rapamycin; p70S6K, p70S6 kinase; CQ, chloroquine; SD, standard deviation.

